# Supplementary material for: Four families of folate-independent methionine synthases
Source: PLoS Genet. 2021 Feb 3;17(2):e1009342. doi: 10.1371/journal.pgen.1009342 (PMC7857596; doi:10.1371/journal.pgen.1009342)
Supplement: S1 Table — (PDF) [file pgen.1009342.s001.pdf]

Supplementary Table S1: The taxonomic distribution of methionine synthases across UniProt's reference proteomes.

Note: Orders that do not core methionine synthases are not shown. Unclassified organisms are not shown.

Domain : Phylum: Class	Order	Proteomes analyzed	How many proteomes contain: MesA	MesB	MesC	MesD/ MesX	MetE	Split MetE	MetH	Genera containing core methionine synthases (up to two)
Archaea : Euryarchaeota										
Archaeoglobi	Archaeoglobales	6			5			1		*Archaeoglobus, Ferroglobus, ...*
Methanobacteria	Methanobacteriales	20	20							*Methanobacterium, Methanobrevibacter, ...*
Methanococci	Methanococcales	5	5							*Methanocaldococcus, Methanococcus, ...*
Methanomicrobia	Methanocellales	2	2							*Methanocella*
Methanomicrobia	Methanomicrobiales	12	12	6						*Methanocorpusculum, Methanoculleus, ...*
Methanomicrobia	Methanosarcinales	16			16					*Methanoperedens, Syntrophoarchaeum, ...*
Methanopyri	Methanopyrales	1	1							*Methanopyrus*
Thermoplasmata	Methanomassiliicoccales	1		1						*Methanomassiliicoccus*
Bacteria : Actinobacteria										
Actinobacteria	Corynebacteriales	182				5	121		151	*Dietzia, Gordonia, ...*
Actinobacteria	Kineosporiales	6				2	3		1	*Quadrisphaera*
Actinobacteria	Micrococcales	273				58	152		121	*Agreia, Amnibacterium, ...*
Bacteria : Bacteroidetes										
Chitinophagia	Chitinophagales	62				1	47		55	*Chitinophaga*
Cytophagia	Cytophagales	102				1	40		100	*Chryseolinea*
Flavobacteriia	Flavobacteriales	248				11	54		232	*Algibacter, Avrilella, ...*
Sphingobacteriia	Sphingobacteriales	89				4	64		70	*Mucilaginibacter, Pedobacter*
Bacteria : Chloroflexi										
Dehalococcoidia	Dehalococcoidales	1		1						*Dehalococcoides*

Supplementary Table S1: The taxonomic distribution of methionine synthases across UniProt's reference proteomes.

Domain : Phylum: Class	Order	Proteomes analyzed	How many proteomes contain: MesA	MesB	MesC	MesD/ MesX	MetE	Split MetE	MetH	Genera containing core methionine synthases (up to two)
Bacteria : Firmicutes										
Clostridia	Clostridiales	126		**29**			73	1	91	*Desulforudis, Carboxydocella, ...*
Clostridia	Halanaerobiales	1		**1**					1	*Frackibacter*
Clostridia	Thermoanaerobacterales	12		**9**			5			*Ammonifex, Calderihabitans, ...*
Negativicutes	Selenomonadales	7		**7**					7	*Anaerospora, Sporomusa*
Bacteria : Fusobacteria										
Fusobacteriia	Fusobacteriales	11		**1**			3		10	*Fusobacterium*
Bacteria : Nitrospirae										
Nitrospira	Nitrospirales	12		**4**			3		8	*Magnetobacterium, Magnetoovum, ...*
Bacteria : Planctomycetes										
Brocadiae	Brocadiales	7		**7**						*Brocadia, Jettenia, ...*
Bacteria : Proteobacteria										
α-Proteobacteria	Caulobacterales	27				**1**	10		27	*Asticcacaulis*
α-Proteobacteria	Rhizobiales	277				**16**	53		268	*Agrobacterium, Aquamicrobium, ...*
α-Proteobacteria	Rhodobacterales	137				**10**	13		132	*Ketogulonicigenium, Paracoccus, ...*
α-Proteobacteria	Rhodospirillales	105				**20**	34		102	*Acetobacter, Asaia, ...*
α-Proteobacteria	Sphingomonadales	189				**21**	19		184	*Aestuariisphingobium, Altererythrobacter, ...*
β-Proteobacteria	Burkholderiales	290				**29**	182		268	*Achromobacter, Advenella, ...*
β-Proteobacteria	Neisseriales	43				**1**	39		20	*Vitreoscilla*
β-Proteobacteria	Nitrosomonadales	49				**1**	37		48	*Nitrosomonas*
γ-Proteobacteria	Aeromonadales	8				**4**	4		8	*Oceanimonas, Oceanisphaera*

Supplementary Table S1: The taxonomic distribution of methionine synthases across UniProt's reference proteomes.

Domain : Phylum: Class	Order	Proteomes analyzed	How many proteomes contain: MesA	MesB	MesC	MesD/ MesX	MetE	Split MetE	MetH	Genera containing core methionine synthases (up to two)
γ-Proteobacteria	Alteromonadales	104				39	44		98	*Alishewanella, Alteromonas, ...*
γ-Proteobacteria	Cardiobacteriales	4				1	3			*Suttonella*
γ-Proteobacteria	Cellvibrionales	27				3	11		27	*Cellvibrio, Microbulbifer*
γ-Proteobacteria	Chromatiales	61				4	29		58	*Rheinheimera*
γ-Proteobacteria	Enterobacterales	126				49	118		89	*Biostraticola, Buttiauxella, ...*
γ-Proteobacteria	Oceanospirillales	104				31	56		101	*Amphritea, Bacterioplanes, ...*
γ-Proteobacteria	Pasteurellales	27				3	25		10	*Bibersteinia, Conservatibacter, ...*
γ-Proteobacteria	Pseudomonadales	92				35	54		83	*Acinetobacter, Alkanindiges, ...*
γ-Proteobacteria	Thiotrichales	28				1	14		28	*Methylophaga*
γ-Proteobacteria	Vibrionales	51				24	43		46	*Enterovibrio, Photobacterium, ...*
γ-Proteobacteria	Xanthomonadales	90				5	28		89	*Pseudoxanthomonas, Stenotrophomonas, ..*
δ-Proteobacteria	Desulfarculales	3		3						*Desulfarculus, Desulfocarbo, ...*
δ-Proteobacteria	Desulfobacterales	25		24			1		2	*Electronema, Electrothrix, ...*
δ-Proteobacteria	Syntrophobacterales	8		7			1		1	*Desulfacinum, Desulfobacca, ...*
Bacteria : Spirochaetes										
Spirochaetia	Brachyspirales	1		1						*Brachyspira*
Bacteria : Thermodesulfobacteria										
Thermodesulfobacter	Thermodesulfobacteriale	4		4						*Caldimicrobium, Thermodesulfatator, ...*
Bacteria : Verrucomicrobia										
Verrucomicrobiae	Verrucomicrobiales	10				1	6		10	*Rubritalea*
