# Supplementary material for: Four families of folate-independent methionine synthases
Source: PLoS Genet. 2021 Feb 3;17(2):e1009342. doi: 10.1371/journal.pgen.1009342 (PMC7857596; doi:10.1371/journal.pgen.1009342)
Supplement: S1 Fig — (PDF) [file pgen.1009342.s002.pdf]

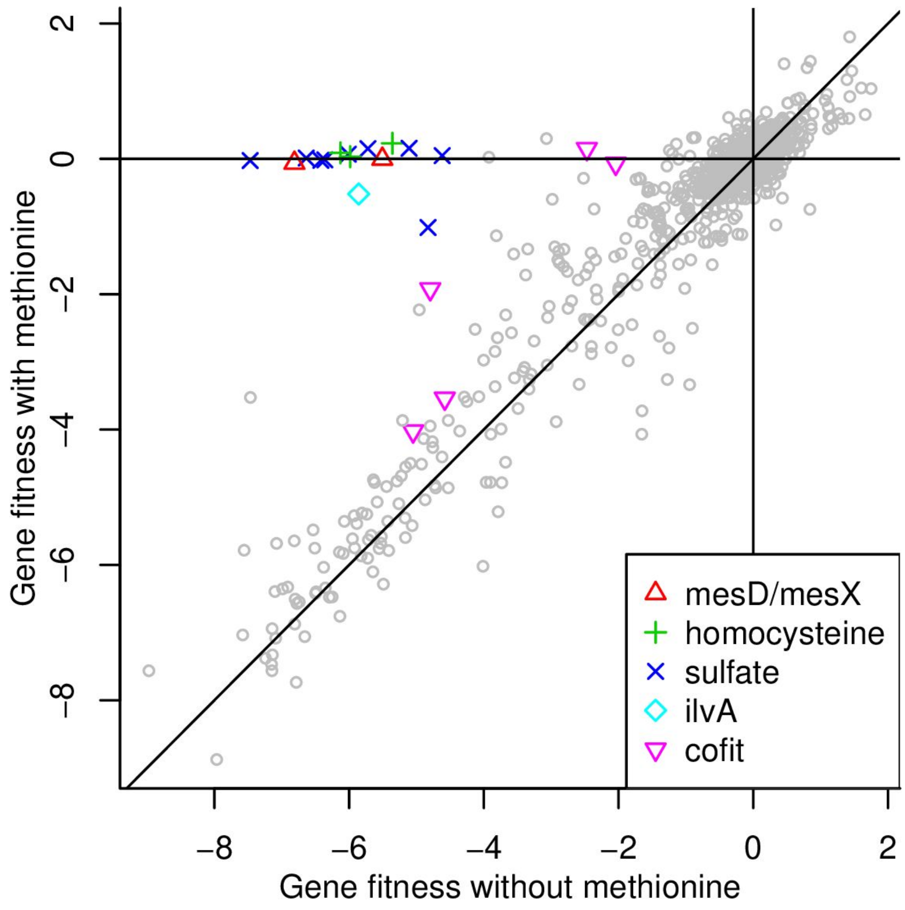

Supplementary Figure S1: Gene fitness from Sphingomonas koreensis growing in minimal glutamate media with or without methionine. We highlight *mesD*, *mesX*, genes involved in homocysteine biosynthesis or sulfate assimilation, *ilvA*, and a few other genes that were cofit with *mesD* and were more important for fitness in the absence of methionine. Sulfate is the sulfur source in our defined medium for *S. koreensis*, but sulfate assimilation is not necessary if methionine is available. Mutants in the threonine ammonia-lyase *ilvA* (Ga0059261_3694), which is the first step in isoleucine biosynthesis, were also rescued by methionine, probably because IlvA's product (2-oxobutanoate) can instead be formed by methionine gamma-lyase (Ga0059261_1458). We also highlight five other genes that were less important for fitness when methionine was added and were cofit with *mesD.* (We defined cofit as having a Pearson correlation of 0.74 or above, which placed them in the top 20 genes.) These genes included the global regulator *dksA* (Ga0059261_0274), genes involved in cell wall biosynthesis (Ga0059261_1360, Ga0059261_2021), a putative sulfur dioxygenase (Ga0059261_2260), and a member of the uncharacterized ApaG family (Ga0059261_3195). The putative dioxygenase has mild phenotypes in many conditions where *mesD* is very important for fitness, so we do not expect that it is directly involved in methionine biosynthesis by MesD/MesX. ApaG (Ga0059261_3195) is more cofit with sulfate assimilation genes such as Ga0059261_4041 (*cysNC, r* = 0.87) than with *mesD* (*r* = 0.76).
