# Supplementary material for: Four families of folate-independent methionine synthases
Source: PLoS Genet. 2021 Feb 3;17(2):e1009342. doi: 10.1371/journal.pgen.1009342 (PMC7857596; doi:10.1371/journal.pgen.1009342)
Supplement: Attachment — Submitted filename: responses.pdf [file pgen.1009342.s003.pdf]

Comments from Reviewer #1

Comment: In the Methods, I noted that HMMER3 searches were reported to use --cut_tc as a command line switch. For future work, --cut_ga would be better, if the two are different, since --cut_tc is often set to the lowest score actually observed above the gathering threshold cutoff, rather than lowest score that would be accepted as valid hit - that's how Pfam does it.

Response: For all of the analyses we ran with curated HMMs, the gathering cutoff (--cut_ga) and the trusted cutoff (--cut_tc) give identical results. The two cutoffs are the same for PF08267.12 (Meth_synt_1), PF01717.18 (Meth_synt_2), PF02219.17 (MTHFR), TIGR01371 (MetE), and TIGR02082 (MetH). For PF08908.11 (DUF1852), the two cutoffs are different, but there are no sequences that score between them in UniProt reference proteomes. In the revised Methods, we briefly mention that the gathering cutoff gives the same results.

Comments from Reviewer #2

Comment: (Introduction) The authors introduce different types of methionine synthases and outline the key findings of their work. Nevertheless, the introduction misses the formulation of the knowledge gap that this paper is going to fill. Please clearly state the problem or lack of knowledge regarding the core methionine synthases, which is tackled in this work. Please also relate to the problem formulation in the Results and Discussion part if possible.

Response: We added a paragraph to the Introduction to summarize the knowledge gaps: "Homologs of these core methionine synthases are found in diverse bacteria and archaea, but it is not clear if these homologs have the same functions. And even for the characterized enzymes, the source of the methyl groups are not known. Furthermore, as we will explain, some organisms that grow in minimal media do not contain any of the known forms of methionine synthase."

Each of the sections in the Results and Discussion has a clear relationship to these knowledge gaps, so we did not modify them.

Comment: This work well presents the differences between known methionine synthases, particularly regarding the cofactor usage, and relates the findings to different domains or phyla. Is it just convergent evolution finding different ways of accomplishing the same objective (synthesis of methionine), or are their implications of picking one cofactor vs another? What would be the advantage of different cofactors being chosen by different evolutionary paths or may the “choice” be caused by differences in the environmental niches? I advise to elaborate on this further in the Discussion part.

Response: The existence of three distantly-related families of corrinoid-dependent methionine synthases does suggest convergent evolution. But it is also conceivable that the very earliest cells used core methionine synthases, and that MetE evolved later (as suggested by Deobald et al 2020). We prefer not to speculate about these ancient events.

In regards to the advantage of different cofactors -- our submission did speculate that "MesD/MesX co-occurs with the other methionine synthases because MesD/MesX is only active under aerobic conditions". We added the following sentence afterwards: "On the other hand, MesD/MesX has the advantage of not requiring cobalamin (or other cobamides) for activity."

For the other core methionine synthases, MesA, MesB, and MesC -- we added a sentence to the

Conclusions about how we see their role. "These core methionine synthases may provide a shortcut from central metabolism to methionine: instead of transferring methyl groups from a corrinoid protein to tetrahydrofolate and then to back to another corrinoid protein (namely MetH), it is simpler to transfer the methyl group directly from MtrA or CoFeSP."

Comment: I ask the authors to comment on future research subjects, experiments etc. to verify the hypotheses stated in this work.

Response: We added a sentence to the Conclusions -- "We hope that biochemical studies will test our predictions that MesA and MesB use corrinoid proteins (MtrA and CoFeSP, respectively) as methyl donors; that MesC proteins are methionine synthases; that MesX uses a flavin cofactor; and will identify the substrate of MesX."

Comment: (Line 99ff) At this point, it is not clear (yet) if the definition of families among the core methionine synthases is a result of this work or was adapted from literature. Please revise.

Response: We realized that we had used the term "families" earlier in the Introduction, which might have misled the reviewer. In our revision, we use other terms instead. The additional paragraph in the Introduction should also help clarify this.

Comment: Co-occurrence of corrinoid genes with particular methionine synthase genes are the primary evidence for their cofactor use it seems – to what extent has this been experimentally validated?

Response: As mentioned in the Introduction, MesA and MesB use methylcobalamin as a substrate in vitro. Their physiological substrates are thought to be corrinoid proteins, but we are not aware of any direct experimental evidence. Based on co-occurence, we proposed that MesA uses MtrA as a substrate, while MesB and MesC use CoFeSP as a substrate. In the revised Conclusions, we mention the need for biochemical tests of these hypotheses.

Comment: (Line 314ff) Can the compatibility of the amino acid change from Glu665 to ASP with zinc binding or coordination be connected to a (or another) zinc-finger motif? Is there a connection between known zinc-finger motifs and the zinc binding residues? Please comment on this, also regarding the mentioned exception in the following paragraph.

Response: Since glutamate and aspartate both have carboxylate groups, we don't think it's surprising that this substitution is compatible with zinc binding.

We don't think there's any connection between zinc finger motifs and MetE or core methionine synthases. Zinc fingers typically coordinate a zinc atom with four cysteine/histidine residues. In contrast, MetE/core synthases use two cysteines, a histidine, and a carboxylate group from glutamate or aspartate. Also, we don't think that zinc finger protein are evolutionarily related to MetE -- for instance, they are classified as different folds in SCOPe.

Regarding MesB proteins with asparagine instead of Cys726 (the exception in the following paragraph) -- although it's rare, there are a few zinc-binding proteins that use asparagine as one of the coordinating ligands, and we added a reference to this effect. Similarly, a few zinc-binding proteins use tyrosine (which is found in a few MesC proteins) as a coordinating ligand, and we added a reference for that as well.

Comment: Figure 6: Please clearly state here that the mutants from the Keio collection lack metE and metF, respectively (e.g., include ΔmetE in the headings of the subfigures).

Response: We modified the headings.

Comments from Reviewer #3

Comment: I can only recommend to include additional table (or a figure) that will summarize the distribution of all four identified methionine synthase isozymes across the prokaryotic organisms. it might be a species or higher rank taxa list or a tree on author`s discretion. Figure 3 already shows this distribution at the genome/strain resolution, however only for a subset of taxa that possess the WL pathway. Thus, the proposed summary table may show it at the higher level, e.g. by including the names of respective taxonomic groups (phyla or orders) and representative species that possess the MS isozymes.

Response: We added supplementary table S1, which shows the distribution of the core methionine synthases at the order level and includes examples of genera from each order that contain core methionine synthases.
